# Mobilization of hematopoietic stem cells as a result of innate immunity-mediated sterile inflammation in the bone marrow microenvironment—the involvement of extracellular nucleotides and purinergic signaling

**DOI:** 10.1038/s41375-018-0087-z

**Published:** 2018-03-05

**Authors:** Mariusz Z Ratajczak, Mateusz Adamiak, Monika Plonka, Ahmed Abdel-Latif, Janina Ratajczak

**Affiliations:** 1 Stem Cell Institute at James Graham Brown Cancer Center, University of Louisville, Louisville, KY USA; 2 Department of Regenerative Medicine, Center for Preclinical Research and Technology, Warsaw Medical University, Warsaw, Poland; 3 Division of Cardiovascular Medicine, Gill Heart Institute, University of Kentucky, Lexington, KY USA

**Keywords:** Stem cells, Medical research

## Abstract

Hematopoietic stem/progenitor cells (HSPCs) circulate in peripheral blood (PB) under normal conditions and their number increases in response to stress, inflammation, tissue/organ injury, and may increase up to 100-fold after administration of mobilization-inducing drugs. Mounting evidence suggests that mobilizing agent-induced mobilization of HSPCs from bone marrow into PB is a result of innate immunity-mediated sterile inflammation in the bone marrow (BM) microenvironment. A critical initiating role in this process is played by tissue/organ injury-mediated or pharmacologically induced release from bone marrow-residing granulocytes and monocytes of (i) danger-associated molecular patterns (DAMPs), (ii) reactive oxygen species (ROS), and (iii) proteolytic and lipolytic enzymes. All these factors together trigger activation of the complement and coagulation cascades, both of which orchestrate egress of HSPCs into BM sinusoids and lymphatics. Recent evidence also indicates that, in addition to attenuation of the SDF-1–CXCR4 and VLA-4–VCAM-1 retention axes in the BM microenvironment and the presence of a mobilization-directing phosphosphingolipid gradient in PB, an important role in the mobilization process is played by extracellular nucleotides and purinergic signaling. In particular, a new finding by our laboratory is that, while extracellular ATP promotes mobilization of HSPCs, its derivative, adenosine, has the opposite (inhibitory) effect.

## Introduction

Hematopoietic stem/progenitor cells (HSPCs) circulate in peripheral blood (PB) under normal conditions following circadian rhythm of circulation and their number increases in response to stress, inflammation as well as tissue/organ injury. The number of HSPCs in PB may increase up to 100-fold after administration of drugs that induce mobilization [1–7]. Based on this, the pharmacological mobilization of HSPCs has been exploited since several years as a convenient strategy to obtain these cells for hematopoietic reconstitution after hematopoietic transplant [6, 7]. The obvious advantage of this strategy is that HSPCs mobilized into PB are relatively easily accessible and they engraft fast after transplantation. Several potential mechanisms have been proposed to regulate mobilization, but still more work is needed to shed more light on this process. Therefore, a better mechanistic insight will help to develop more efficient strategies to obtain these cells for clinical purposes. Our groups since several years are studying a role of innate immunity in this process [8–13].

HSPCs are retained in their niches in the bone marrow (BM) microenvironment due to retention signals involving mainly interaction of the CXCR4 and VLA-4 receptors present on their surface with the corresponding ligands, stromal-derived factor 1 (SDF-1), and vascular cell adhesion molecule 1 (VCAM-1), respectively, which are expressed in BM stem cell niches [1, 2]. The importance of both retention axes is supported by the fact that blockade of either CXCR4 or VLA-4 by small-molecule antagonists triggers rapid mobilization of HSPCs into PB [3, 4]. Mobilization of HSPCs into PB is also triggered in response to strenuous exercise, tissue/organ injury, and administration of certain cytokines (granulocyte mobilizing factor, G-CSF) or chemokines (growth-regulated protein beta, Gro-beta) [4–7].

Evidence has accumulated that, in all of these cases, the mobilizing agent induces a cascade of events in the BM microenvironment that can be considered as an example of “sterile inflammation.” According to the definition, sterile inflammation is an inflammatory process that occurs in a given tissue in the absence of any microorganisms [8]. However, like microbial-induced inflammation, sterile inflammation is marked by the activation of cellular and soluble elements of innate immunity, including neutrophils and macrophages as well as the complement cascade (ComC) [8, 9]. In the first step of sterile inflammation, activated granulocytes and monocytes residing in the BM microenvironment release danger-associated molecular pattern (DAMPs) molecules, reactive oxygen species (ROS), proteolytic and lipolytic enzymes, and several pro-inflammatory cytokines and chemokines [8–12]. Mediators released during sterile inflammation, such as DAMPs and ROS, activate ancient enzymatic proteolytic cascades in the BM microenvironment, mainly the complement cascade (ComC) [8, 11] but in addition also the coagulation cascade (CoaC) [13–15]. Mice deficient in some  elements of the ComC (e.g., C5) are poor mobilizers of HSPCs [16, 17]. Clinical data also support an important role for ComC activation during mobilization in patients [18].

Induction of sterile inflammation in BM is crucial for (i) release of HSPCs from their niches, (ii) permeablization of the BM–PB endothelial barrier, and (iii) egress of neutrophils and monocytes into PB in a process that paves the way for HSPCs to follow the mobilizing gradient of bioactive phosphosphingolipids (sphingosine-1-phosphate, S1P, and ceramide-1-phosphate, C1P) originating in PB [19–21]. Egress of HSPCs into lymphatics is also directed by S1P and C1P [22].

The crucial role of S1P and C1P in the egress of HSPCs is supported by the fact that both of these phosphosphingolipids create strong chemotactic gradients for HSPCs across the BM–PB endothelial barrier already  under steady-state conditions [19]. The retention of HSPCs in BM niches also indicates an active retention process for HSPCs that counteracts these gradients. Furthermore, evidence has accumulated that mobilization of HSPCs correlates with the level of S1P in PB and is impaired in mice that have low levels of S1P in PB due to sphingosine kinase 1 deficiency [20] and are enhanced in mice with sphingosine 2 kinase deficiency, which, somewhat surprisingly, have elevated levels of S1P in PB plasma [23].

In this editorial, we will present the mounting body of evidence that an important trigger of sterile inflammation in BM is the release of DAMPs and ROS from activated Gr-1^+^ granulocytes and monocytes. Both DAMPs and ROS are major activators of the ComC by involving the mannan-binding lectin (MBL) pathway of ComC activation [11]. The crucial DAMPs in this process are extracellular nucleotides, such as ATP, which are released by pannexin hemichannels expressed, e.g., on the cell membranes of granulocytes and monocytes [8, 11]. Also, contributing to this process are ROS released from these cells, and in the BM microenvironment ROS-exposed neo-epitopes bind naturally occurring antibodies (NAbs) [24]. Both DAMPs and neoepitope–NAb complexes are recognized by MBL, a soluble member of the class of innate immunity elements known as pattern recognition receptors (PRRs) [8, 9, 11].

### Activation of granulocytes and monocytes initiates sterile inflammation in BM and triggers the mobilization of HSPCs

**Fig. 1:**
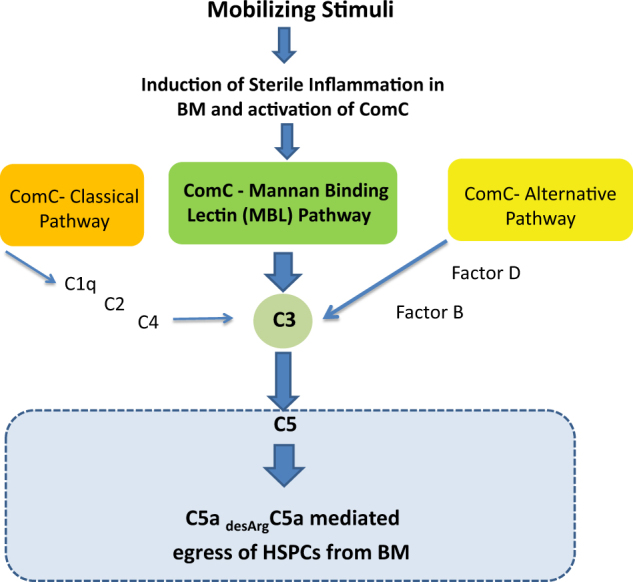
The ComC plays an important role in triggering the mobilization of HSPCs in a mannan-binding lectin (MBL) pathway-dependent manner. Promobilizing stimuli activate sterile inflammation in the BM microenvironment and activation of the complement cascade (ComC), which leads to C5 cleavage and the generation of C5a and _desArg_C5a anaphylatoxins. Both of these C5 cleavage fragments facilitate egress of HSPCs into PB in response to S1P and C1P gradients. Of the three ComC activation pathways (classical, alternative, and mannan-binding lectin, MBL), the MBL pathway plays as we reported [11] a crucial role in the effect of sterile inflammation on the BM microenvironment and activation of the ComC

**Fig. 2:**
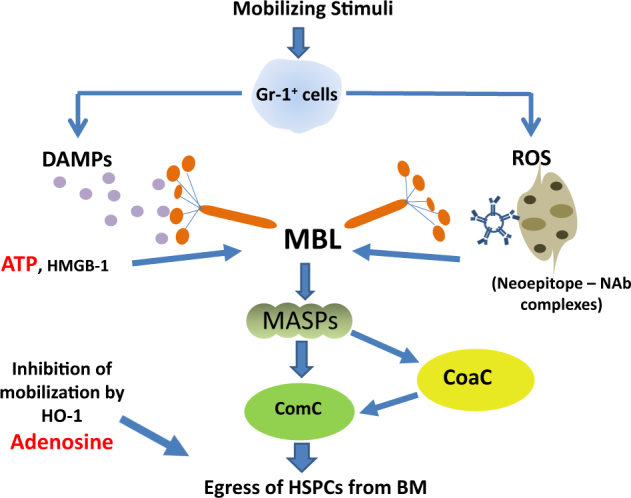
Proposed MBL-induced model for triggering sterile inflammation in BM. A mobilizing agent (e.g., G-CSF) induces secretion of DAMP molecules (e.g., ATP) and ROS from Gr-1^+^ cells (granulocytes or monocytes). ATP directly and ROS indirectly (by exposing neoepitope antigens in the BM microenvironment that are recognized by naturally occurring antibodies [Nabs] to form neoepitope–NAbs complexes) are recognized by MBL, which initiates activation of the ComC in a MBL-MASPs-dependent manner. The C5 cleavage fragments, anaphylatoxins C5a and _**desArg**_C5a, facilitate egress of HSPCs from BM into PB by augmenting further degranulation of granulocytes and attenuating BM-retention axes in the stem cell  niches (by release of proteolytic and lipolytic enzymes) and chemoattracting granulocytes and monocytes into PB to pave the way for HSPC migration across the BM–PB barrier. As a result, HSPCs released from their niches follow the S1P and C1P gradients originating in PB. In parallel, MASP-1 also activates the CoaC. Egress of HSPCs from BM into PB is negatively regulated by the anti-inflammatory action of HO-1 and adenosine. In red color is highlighted ATP released from Gr-1^+^ cells as DAMP—that promotes mobilization and its degradation product adenosine—that has an opposite effect on mobilization process

A mounting body of evidence demonstrates the crucial involvement of the ComC as well as granulocytes and monocytes in triggering the mobilization of HSPCs (Fig. 1). The pivotal role of Gr-1^+^ granulocytes and monocytes in the mobilization process has already been well demonstrated in elegant studies performed in mice [25, 26]. These cells are important cellular components of innate immunity and are activated in the BM microenvironment by promobilizing signals released from damaged tissues as well as by pharmacological agents employed for mobilization of HSPCs. As depicted in Figs. 1 and 2, they are also first-responder cells in initiating sterile inflammation by secreting several proteolytic and lipolytic enzymes [27, 28] and—what is important for the topic of this review—also DAMPs and ROS. While a role for proteolytic enzymes in attenuating SDF-1–CXCR and VCAM-1–VLA interactions between HSPCs and their niches has been proposed by other groups [1, 2, 27], our recent evidence indicates that the lipolytic enzyme phospholipase C β2 (PLC-β2), which is released from granulocytes and digests glycosylphosphatidylinositol anchor (GPI-A) in cell membranes, plays a crucial role in the release of HSPCs from BM niches [28]. To explain this effect, CXCR4 and VLA-4 expressed on HSPCs are incorporated into membrane lipid rafts for optimal function [29], and by digesting GPI-A, PLC-β2 destroys the integrity of cell surface membrane lipid rafts and thus attenuates HSPC retention in BM [28]. Cells released from BM niches become susceptible to chemotactic gradients of S1P and C1P that are present in BM sinusoids [19–21].

However, according to the main point of this review, the first step in the induction of sterile inflammation and activation of the ComC is the release of DAMPs by granulocytes and monocytes [8, 11, 30–33]. The most important DAMPs released from these cells upon their activation by mobilizing cues are ATP [8, 11] and high group mobility box 1 (HGMB-1) protein [8, 30]  ATP is secreted through cell surface pannexin hemichannels [8, 11, 30]. In support of this mechanism, we observed that inhibition of pannexin 1 channels by probenecid results in attenuation of the mobilization of HSPCs (manuscript in preparation). It has also been reported that two other mechanisms are responsible for DAMP release, one involving connexin 43 and the other involving extracellular microvesicles (ExMVs) [8]. Interestingly, mice deficient in connexin 43 have been reported to be poor mobilizers [34], and a potential role of connexin 43 in ATP release may additionally be responsible for the poor mobilization status of these mice.

ATP is a well-known ubiquitous intracellular molecular energy source, but in addition it may also be secreted into the intercellular space, where it acts as an important signaling molecule in purinergic signaling [35]. In contrast to ATP, HGMB-1 is just a nuclear protein that binds DNA in a non-sequence-specific manner and is involved in chromatin remodeling and bending [8]. As mentioned above, both ATP and HGMB-1 are recognized by a member of the class of soluble PRRs, MBL [8, 11, 30–33]. After binding to DAMPs, MBL activates the MBL-associated serine proteases MASP-1 and -2, which initiate activation of the ComC (Figs. 1 and 2). Of the three ComC activation pathways—classical, alternative, and MBL—the last plays as we reported a pivotal role in the induction of sterile inflammation in BM and in triggering the mobilization of HSPCs. In support of this mechanism, we recently reported that mice deficient in MBL (MBL-KO mice) are poor mobilizers of HSPCs [11]. By contrast, mice deficient in elements of the classical pathway of ComC activation, such as C1q-KO mice, are easy mobilizers [36]. Our indirect results also indicate that a supportive role in the mobilization process is played by the alternative pathway, as mice deficient in factor B (an element of the alternative pathway of ComC activation) also show a defect in the mobilization process [37].

In parallel to DAMPs, activated granulocytes and monocytes also release ROS, which, as mentioned above, by oxidation of cell membranes on cells in the BM microenvironment, expose neo-epitopes that are recognized by NAbs of the IgM class. NAbs are produced without any previous infection, vaccination, or other foreign antigen exposure and are important soluble components of innate immunity [9, 24]. Their physiological role is restricted to acting as key regulators in recognizing neo-epitopes exposed on the surface of damaged cells. As previously demonstrated, such neoepitope–NAbs complexes are, like DAMPs, recognized by MBL [24, 31] and activate the ComC via MASP activation (Fig. 2).

Of the two MASPs present in serum (MASP-1 and -2), MASP-1 seems to play a crucial role in activating the ComC [32]. In addition, MASP-1 is also involved in activation of the CoaC by activating prothrombin to thrombin, which explains why both cascades are activated in parallel during the mobilization process [13–15]. Moreover, it has been reported that, during the coagulation process, thrombin exhibits C5 convertase-like activity [38]. Therefore, the evidence indicates that coordinated crosstalk between both cascades potentiates activation of the distal part of the ComC by cleavage of C5 into the bioactive C5a and _desArg_C5a anaphylatoxins. In case of a deficiency of the proximal C3 complement component, thrombin C5 convertase-like activity may also replace the function of classical C5 convertase activity provided by activated C3 cleavage fragments [13]. This demonstrates the presence of redundancy in supplying C5 convertase activity by both the ComC (in its classical form) and the CoaC (in its “activity-like” form). Further supporting this involvement of the CoaC in the mobilization process, exposure of mice to the thrombin inhibitor heparin has a negative effect on G-CSF- or AMD3100-induced egress of HSPCs from BM into PB [13]. This is not surprising, given that the inflammation and coagulation processes are activated in parallel by similar stimuli [8, 9].

Based on this mechanism, the final step of sterile inflammation in the BM microenvironment and in activation of the ComC and CoaC during the mobilization of HSPCs is cleavage of C5 into C5a and _desArg_C5a. In fact, mice that are deficient in C5 are poor mobilizers [16]. This strongly supports a requirement for C5 cleavage and the generation of two important anaphylatoxins, C5a and _desArg_C5a, in the egress of HSPCs from BM into PB [16]. What is also interesting, mice that are deficient in the C5 component of the ComC do not also display circadian changes in circulation of HSPCs in blood [39]. The open question is whether this circadian release of HSPCs reflects induction of circadian peaks of sterile inflammation in the BM microenvironment due to deep-sleep hypoxia [39]. In fact, circadian activation of the ComC has been demonstrated by other investigators [40].

### The emerging role of extracellular nucleotides and purinergic signaling in initiating sterile inflammation in the BM microenvironment

As mentioned above, ATP secreted from G-CSF-activated neutrophils and monocytes is a crucial DAMP molecule involved in induction of sterile inflammation in BM during the mobilization of HSPCs, leading to ComC activation in an MBL-dependent manner [11]. However, besides serving as a DAMP ligand for MBL, ATP is also involved as part of purinergic signaling in initiating several other pathways controlling release of HSPCs from BM into PB that are still not completely understood [35]. Biological effects of ATP and other extracellular nucleotides as part of purinergic signaling in BM have been reported for normal and leukemic cells [41, 42]. In addition to hematopoietic cells, extracellular nucleotides and purinergic signaling also modulate the function of other BM components, including mesenchymal cells and endothelial cells [41]. Thus, in addition to the relatively well-studied effects of peptide-based growth factors, cytokines, and chemokines, small-molecule extracellular nucleotides add significant diversity to the collection of known players in sterile inflammation and are necessary to better understand the development of pre-natal [43] and post-natal hematopoiesis [41] as well as stem cell trafficking [41, 42].

Overall, purinergic signaling is an evolutionarily ancient signaling mechanism regulating several aspects of cell biology, such as the generation of chemotactic signals and modulation of the responsiveness of innate and acquired immune cells to inflammatory cues [35]. Purinergic signaling is involved in homeostasis of the organism and regulates neurotransmission, cardiac and vascular function, platelet aggregation, and hematopoiesis [35]. These pleiotropic functions are regulated by ATP and its metabolites, such as ADP, AMP, and the nucleoside adenosine, as well as by other nucleotides such as the pyrimidine nucleotides UTP, UDP, and certain nucleotide derivatives like UDP-glucose [35, 41]. After many years of skepticism, purinergic signaling is now widely acknowledged, and several cell surface receptors have been cloned and shown to be expressed on cells in almost every tissue in the body [35, 41].

On the surfaces of hematopoietic cells we can distinguish the expression of both nucleotide- and nucleoside-activated receptors, which belong to two different purinergic receptor families, P2 and P1 [41]. P2 receptors are further subdivided into metabotropic (P2Y) and ionotropic channel (P2X) receptors based on structural characteristics [35, 41]. The P2Y receptor family, including eight receptors that have been identified so far (P2Y1, 2, 4, 6, 11, 12, 13, and 14), are G protein-coupled receptors and respond to stimulation by ATP, ADP, UTP, UDP, and UDP-glucose [41]. The P2X ionotropic channel receptor family consists of seven members (P2X1, 2, 3, 4, 5, 6, and 7), which are activated by ATP, opening the channel to allow an influx of Ca^2+^, Na^+^, and K^+^[35, 41]. The P1 receptor family consists of four G protein-coupled receptor subtypes, A_1_, A_2A_, A_2B_, and A_3_, which are activated by adenosine and its analogs [35, 41].

This large number of receptors and ligands shows the complexity of the purinergic signaling system. It also indicates that ATP, being a major DAMP molecule secreted by activated neutrophils and monocytes and a trigger for MBL-mediated activation of the ComC, may also affect several other responses in cells within the hematopoietic microenvironment by interacting with the corresponding purinergic receptors [41]. Furthermore, processing of ATP in the extracellular space by ectonucleotidases such as CD39 and CD73, leading to generation of the ATP metabolites ADP, AMP, and adenosine, generates new bioactive signaling molecules in the extracellular space [41, 44]. Also present in the extracellular space are other mediators of purinergic signaling, such as UTP and UDP [35, 41, 42]. In addition to the DAMP activity of ATP, evidence suggests that there are effects of extracellular nucleotides in mobilization that are not directly mediated by ATP. For example, mice exposed to UDP-glucose, which directly activates the P2Y14 receptor, mobilize long-term repopulating hematopoietic stem cells into peripheral blood, and animals that are P2Y14 deficient display accelerated senescence of HSPCs in response to radiation stress, chemotherapy, and the normal physiological aging process [44].

Interestingly, our most recent results indicate that, while injection of ATP enhances the mobilization of HSPCs, its final metabolite adenosine has the opposite effect (manuscript in preparation). Adenosine is well known for its immunosuppressive and anti-inflammatory effects [45]. Given that mobilization of HSPCs requires the induction of sterile inflammation in the BM microenvironment, an anti-inflammatory effect of adenosine explains this inhibitory phenomenon. Moreover, ATP is released by cells at much higher concentrations than UDP-glucose, which indicates a more important role for this extracellular nucleotide in the mobilization process [8, 44]. Moreover, since ATP does not interact with the P2Y14 receptor [44], this suggests that ATP may influence the mobilization process through means other than its DAMP activity by interacting with other receptors of the purinergic signaling family.

More work is also needed to phenotype BM stem cell niches for the expression of purinergic receptors. However, the hematopoietic stem cell niche remains incompletely defined and is described by competing models [46, 47]. Recent research indicates that this niche is perivascular (SDF-1^+^ and KL^+^), created partially by mesenchymal stromal cells and endothelial cells, and is often, but not always, located near trabecular bone [46]. While HSCs are located around perivascular cells, it has been proposed that early lymphoid progenitors are associated with the osteoblastic niche [46]. Moreover, the existence of distinct niches for distinct subpopulations of HSCs, including quiescent nestin^bright^ NG2^+^ arteriolar and proliferative nestin^dim^Lepr^+^ sinusoidal niches, have also been proposed [46, 47]. Overall, the effect of purinergic signaling in maintaining the integrity of these niches under steady-state conditions and during mobilization requires further study. Furthermore, given the role of osteoclasts in mobilization [48], extracellular nucleotides may affect the biological functions of these cells.

In particular, since it has been proposed that beta-adrenergic signaling is involved in egress of HSPCs into circulation [49] and because  purinergic signaling is involved in addition to catecholamine and acetylcholine in neurotransmission [35], further studies are needed to explain the potential interaction between purinergic signaling and the signaling of other neurotransmitters in the mobilization process. As mentioned above, it will also be interesting to see the effect of extracellular nucleotides on the circadian rhythm of stem cell release from BM into PB.

Based on all of these considerations, the involvement of purinergic signaling in maintaining normal and stress-induced hematopoiesis needs reappraisal. This is a viable task, particularly since additional KO animal models are now available. Moreover, several new and more-specific small-molecule modulators of purinergic receptor family signaling as well as CD39 and CD73 ectonucleotidases that degrade ATP in the extracellular space to its active metabolites, including adenosine, are now available.

### Inhibitors of sterile inflammation as negative regulators of HSPC mobilization

Since the induction of sterile inflammation is crucial in triggering the HSPC mobilization process [11], an anti-inflammatory treatment will have the opposite effect. As mentioned above, the extracellular ATP degradation product adenosine is an important known anti-inflammatory mediator [45], which, as we recently observed, inhibits egress of HSPCs into PB. Since adenosine is generated in the extracellular space by CD73 ectonucleotidase, we expect that CD73-KO mice, which do not process conversion of AMP to adenosine in the extracellular space, should be easy mobilizers.

Another important inhibitor of stem cell mobilization that counteracts BM sterile inflammation is heme oxygenase 1 (HO-1), and one of its demonstrated effects is inhibition of cell migration [50]. HO-1 is an inducible enzyme that is upregulated in the BM microenvironment in response to several oxidative stress stimuli [51–55]. The anti-inflammatory and anti-ComC activation effects of HO-1 have been very well demonstrated in an HO-1-deficient mouse model as well as in a case of human HO-1 deficiency. In both of these cases, the ComC becomes hyperactivated due to the lack of a balancing inhibitory effect by HO-1 [54]. Demonstrating an inhibitory effect of HO-1 on stem cell mobilization, we have reported that (i) HO-1-deficient mice are easy mobilizers of HSPCs [52], and (ii) upregulation of HO-1 in hematopoietic cell lines by employing small-molecule activators decreases cell chemotaxis, while intracellular downregulation of HO-1 has the opposite effect [52]. We observed similar effects when modifying the expression of HO-1 in normal murine and human cells enriched for HSPCs [52, 56].

Based on this logic, inhibition of adenosine generation in the BM extracellular space, for example, by inhibition of the ectonucleotidase CD73 expressed on the surface of BM cells or downregulation of HO-1 by employing small-molecule inhibitors of this enzyme, should promote sterile inflammation in the BM microenvironment, and facilitate egress of HSPCs from BM into PB. Future work is also needed to see how extracellular nucleotide and purinergic signaling affect expression of CD73 and HO-1 in BM and which receptors involved in those effects are potential therapeutic targets.

## Conclusions

We propose a crucial role for innate immunity-mediated sterile inflammation induced in the BM microenvironment by mobilization-inducing stimuli as a major driving force in the egress of HSPCs across the BM–PB endothelial barrier into the BM sinusoids. A parallel process most likely directs the egress of HSPCs into lymphatics.

Recent evidence indicates that the onset of sterile inflammation in BM is tightly connected with an increase in the level of extracellular nucleotides, mainly ATP, which, as a DAMP molecule, activates the ComC in an MBL-dependent manner [11]. This effect does not preclude  an additional role of ATP and its degradation products (ADP, AMP, and adenosine) in a non-DAMP-related modulation of this process. In addition to Gr-1^+^ cells ATP may be also released from other hematopoietic and non-hematopoietic cells in BM upon their activation or in response to damage. The release mechanism involved appears to be based on a combination of vesicular exocytosis and involvement of pannexin and connexin hemichannels. The expression and abundance of functional purinergic receptors expressed on HSPCs and other type of cells in the BM microenvironment including perivascular SDF-1^+^ and KL^+^ mesenchymal stromal cells and endothelial cells as well as cells in quiescent nestin^bright^ NG2^+^ arteriolar and proliferative nestin^dim^Lepr^+^ sinusoidal niches, opens up a new area of investigation to better understand the complexity of this process, which will be crucial in designing more efficient mobilization strategies. Furthermore, given the role of osteoclasts in mobilization [48], extracellular nucleotides may affect the biological functions of these cells as well. More work is also needed to understand a role of purinergic signaling as neurotransmitters in neural fibers in innervating BM tissue [35] and its potential effect on modulation of beta-adrenergic regulation of HSPCs mobilization [49].
